# Adsorptive removal of adsorbable organic halogens by activated carbon

**DOI:** 10.1098/rsos.181507

**Published:** 2018-12-05

**Authors:** Chengrong Qin, Baojie Liu, Lingzhi Huang, Chen Liang, Cong Gao, Shuangquan Yao

**Affiliations:** 1 College of Light Industrial and Food Engineering, Guangxi University, Nanning 530004, People's Republic of China; 2 Guangxi Key Laboratory of Clean Pulp and Papermaking and Pollution Control, Nanning 530004, People's Republic of China

**Keywords:** adsorbable organic halogens, activated carbon, adsorption, volatilization, photodecomposition

## Abstract

Current research mainly focuses on the reduction of adsorbable organic halogen (AOX) sources, while studies on AOX monitoring and management in the environment are scarce. Organic pollutants in water are mainly fixed by sediments. Thus, in this paper, activated carbon was used to simulate the adsorption of AOX by sediments. AOX volatilization and degradation were also studied to exclude their effect on adsorption. Micromolecule chlorides were more easily volatilized and degraded than chlorobenzene and chlorophenol. The adsorption of activated carbon to AOX in bleaching wastewater was also studied and the optimum conditions for AOX removal were elucidated (particle size, 62 µm; time, 120 min; pH, 2.5; temperature, 40°C; and activated carbon dosage, 1.75 g l^−1^). AOX adsorption by activated carbon is a chemical process. Hence, the chemical compositions of the bleaching effluent with and without adsorption were analysed by GC-MS. The results revealed that activated carbon exhibits a good AOX removal effect, thereby providing a theoretical basis for monitoring the AOX distribution in the environment.

## 1. Introduction

Organic pollutants from the pulp and paper industry are mainly attributed to the bleaching process. A large amount of organic chlorides is produced by hypochlorite bleaching and chlorination reactions during the traditional chlorination-alkali treatment-hypochlorite bleaching (CEH) process. Bleaching pollution based on traditional CEH bleaching is becoming an increasingly serious environmental issue [1]. Thus, low pollution and high efficiency bleaching technology, such as elemental chlorine free (ECF) and total chlorine free (TCF) bleaching, is the best way to mitigate this problem [2,3]. The formation of adsorbable organic halogens (AOX) has been greatly reduced during the ECF bleaching process. The main components of these compounds are characterized as persistent organic pollutants (POPs) [4]. Thus, similar to POPs, AOX are bio-accumulative, highly toxic, semi-volatile, display long-distance mobility and form persistent/long-term residues. The rise in bleaching wastewater discharge is contributing towards the accumulation of the total AOX formation in the environment. This increase in environmental pollution [5,6] has attracted the attention of both governments and the public worldwide. Thus, each country has specific regulations on AOX emission limits in bleaching effluents: Switzerland = 0.1–0.2 kg tp^−1^, Finland = 0.2–0.4 kg tp^−1^, Canada = 0.25 kg tp^−1^, America = 0.272 kg tp^−1^ and China = 0.72 kg tp^−1^.

Although recent years have seen an increase in research on AOX, studies have mainly focused on AOX formation mechanisms to reduce AOX in the bleaching process. Most studies assume that the residual lignin in the unbleached pulp is the main source of AOX [7]. Hence, due to its complex chemical structure, lignin has been replaced by lignin models [8,9] and the AOX formation mechanism has been studied via the reaction between the lignin model and chlorine dioxide [10]. These studies have also demonstrated that AOX formation is influenced by hemicellulose [11] and hexenuronic acid (HexA) [12,13]. The reduction of AOX in the bleaching process has been realized by process optimization [14], the use of chemical additives [15,16] and pretreatment [11,17–19]. Adsorption is the most widely used method in the treatment of organic pollutants [20–22]. This method has the advantages of a high removal efficiency, thorough purification, low energy consumption, mature technology and easy promotion [23]. Good environmental and economic benefits have also been achieved. Owing to its large specific surface area and a strong adsorption capacity towards organics and metal ions, activated carbon is suitable for various applications, including water purification [24–26]. The anionic toxic contaminant Congo red has been adsorbed by activated carbon [27]. This has led to the production of highly efficient low-cost materials of large capacity to remove anionic ionizable organic contaminants.

The aim of this work is to study the adsorption effect of organic chlorides in bleaching effluents using activated carbon. AOX volatilization and degradation processes were studied to eliminate their effect on AOX adsorption. The effects of active carbon particle size, pH, temperature, dosage and time on the adsorption capacity were also investigated. Finally, the chemical compositions of the bleaching effluent with and without adsorption were analysed by GC-MS. The results present an important guideline for the analysis and treatment of AOX in the environment.

## 2. Experimental procedure

### 2.1. Materials

Unbleached bagasse pulp was provided by a pulp mill in Guangxi, China (pulp kappa number = 11.54, brightness = 40.51% ISO and viscosity = 1349 ml g^−1^). Activated carbon was purchased from the Analytik-Jena Instrument Company (Jena, Germany). Sodium chlorite, hydrochloric acid, sodium hydroxide, sulfuric acid, potassium iodide and sodium thiosulfate were purchased from Sigma-company (Shanghai, China). All chemicals used in the paper were of analytical grade and used as received.

### 2.2. Chlorine dioxide bleaching

The bleaching reaction was carried out in a water bath kettle. First, 10 g unbleached dry pulp was mixed with pure water in a sealed polyethylene bag [mixture concentration = 10% (w/V)]. The mixture was thoroughly mixed for 5 min at 100 rpm. The pH of the reaction solution was adjusted to 3.5–4 by the addition of sulfuric acid solution. Next, 120 kg tp^−1^ (2.0%) chlorine dioxide solution was added to the mixture at 65°C (reaction time = 60 min). The mixture was kneaded every 5 min during the reaction and sufficient contact between the pulp and chlorine dioxide solution was ensured. Most of the lignin was removed during bleaching. The pulp was washed to neutral by pure water at the end of the reaction. The basic structural lignin units were chlorinated. Organic chloride is produced during the bleaching process. Finally, the reaction solution was collected [11].

### 2.3. AOX content analysis

A multi X2500 AOX analyser (Jena, Germany) was employed to identify the AOX present in the bleaching effluent as follows: the bleaching effluent was passed through an activated carbon column to adsorb the organic chloride. The adsorbed inorganic chloride was then washed with sodium nitrate. The activated carbon column was burned in a combustion furnace and the AOX content was calculated [28].

### 2.4. AOX volatilization

In the experiment, 2 l bleaching effluent was loaded in a glass beaker and then purged thoroughly with sodium nitride (1# encapsulation; 2# exposure placement). Sample solutions were withdrawn at different times during the experiments. The bleaching effluent was collected every 2 days at room temperature to detect the AOX content. It was then extracted with ether three times and subsequently combined with the organic phase. Finally, the AOX content was determined.

### 2.5. AOX photodegradation

About 2 l bleaching effluent was loaded in a glass beaker and purged thoroughly with sodium nitride. Both groups were sealed, one of which was treated without light. The sample solutions were withdrawn at different times during the experiments. The bleaching effluent was collected every 2 days at room temperature. Finally, the AOX content was determined.

### 2.6. Chemical composition analysis of the bleaching effluent

The chemical composition of the reaction solution was analysed by GC-MS (Agilent 6890-5973, USA). The organic chlorides were qualitatively and quantitatively analysed. The solution was concentrated with or without volatilization, degradation and adsorption. The concentrate was extracted by column adsorption and 1 µl of the concentrate was then measured. The detailed operating conditions for GC-MS have been described in previous studies [29,30].

### 2.7. Activated carbon adsorption

About 50 ml bleaching effluent (AOX formation = 25.98 mg l^−1^) was withdrawn and placed in a ground conical flask (100 ml). The amount of activated carbon was controlled and the mixture was sealed and placed in a constant temperature oscillator (amplitude = 118 r min^−1^) to attain oscillatory adsorption. After 30 min, the sample was centrifuged. Finally, 1 ml supernatant was withdrawn and the total amount of AOXs was calculated.

## 3. Results and discussion

### 3.1. AOX volatilization

Natural organic compounds display a degree of volatility. Thus, the volatilization regularity of AOX was studied to avoid its effect on adsorption. The volatilization experiments were carried out in the range 5–45 days.

**Figure 1.**
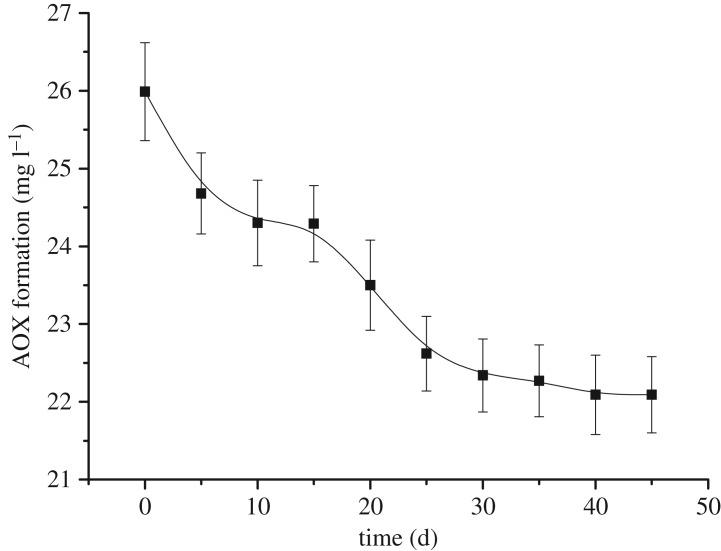
The volatilization of AOX in wastewater.

Figure 1 illustrates the amount of AOX affected by volatilization in 45 days. The amount of AOX decreased from 25.98 mg l^−1^ to 21.97 mg l^−1^ (15.43% decrease), indicating that the volatility of AOX is significant. The results also revealed that the volatility of AOX was the fastest during the first 30 days, with the content decreasing from 25.98 mg l^−1^ to 22.33 mg l^−1^ from day 1 to day 30, respectively (14.40% decrease). This lower value remained constant throughout the rest of the testing period (day 30 to day 45) indicating that no volatile components were present in the AOX during this period. The bleaching effluent comprised four types of organic chlorides (table 1), namely chlorobenzene, chlorophenol, macromolecular organic chloride and micromolecule organic chloride. Chlorobenzene and chlorophenol were the most abundant and unmanageable AOX components (60.80% of the total organic chlorides). On the other hand, the 2,4,6-trichlorophenol, 1,2,4-trichlorobenzene, 2-chlorohydroquinone and chloroacetophenone contents were 0.33%, 0.69%, 0.44% and 0.36% of the total organic chlorides, respectively. Once the AOX content remained stable, i.e. in the absence of volatile components, the sample components were reanalysed. The results revealed a decrease in both the small and large organic chloride contents. This indicated the volatility of some of the chloroacetic acid, 1-bromo-2-chloromethane, 1,1,2,2-tetrachloroethane, 3-chloro-1.2-propanediol, 1,1,3,3-tetrachloroacetone, 2-chloropropionic acid and acetyl chloride. On the other hand, the chlorobenzene and chlorophenol contents remained unchanged. These results confirmed the semi-volatile nature of AOX.

**Table 1.** Analysis of volatile and degradation components.

ingredient	relative content (%)		
	unprocessed	volatilization	degradation
chloroacetic acid	0.22	0.12	0.15
1-bromo-2-chloromethane	0.20	0.16	0.14
1,1,2,2-tetrachloroethane	0.18	0.11	0.13
3-chloro-1.2-propanediol	0.07	/	0.08
2,4,6-trichlorophenol	0.33	0.32	0.33
1,1,3,3-tetrachloroacetone	0.20	0.08	0.12
1,2,4-trichlorobenzene	0.69	0.69	0.69
2-chlorohydroquinone	0.44	0.42	0.43
2-chloropropionic acid	0.14	0.10	0.11
acetyl chloride	0.16	0.09	0.14
chloroacetophenone	0.36	0.36	0.35

### 3.2. AOX photodecomposition

In addition to volatilization, the most important mechanism for the migration and transformation of substances is via degradation. In general, degradation can be divided into two types: photo- and biodegradation. Biodegradation can be ignored under laboratory conditions. Moreover, by studying the photolysis of AOX in the bleaching effluent, the effect of light (400–750 nm) on the experimental results during the adsorption process could also be excluded. Hence, to investigate the effect of time on AOX photodecomposition, photodegradation experiments were carried out at different light exposure times in the range 5–45 days.

**Figure 2.**
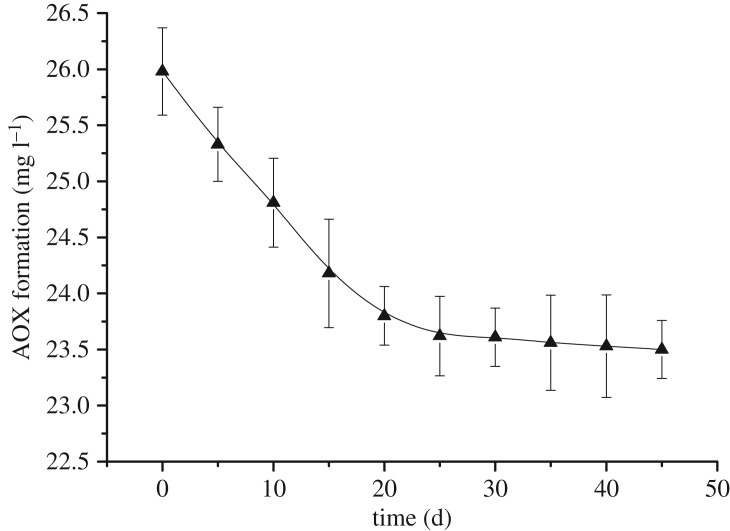
The photolysis of AOX in bleaching effluent.

Figure 2 reveals that the AOX content decreased from an initial 25.98 mg l^−1^ to 23.70 mg l^−1^ (8.78% decrease) after 25 days of photodecomposition. The amount of degradation was stable after 25 days, while the AOX content remained unchanged after 45 days (23.50 mg l^−1^). These results demonstrate that the effect of photodegradation on AOX is limited. Subsequently, when the photolysis of the AOX components became stable, its components were detected. The afforded results (figure 2 and table 1) revealed a decrease in both the micromolecule and macromolecular organic chloride contents, with chloroacetic acid, 1-bromo-2-chloromethane, 1,1,2,2-tetrachloroethane, 3-chloro-1.2-propanediol, 1,1,3,3-tetrachloroacetone, 2-chloropropionic acid and acetyl chloride contents of 0.15%, 0.14%, 0.13%, 0.08%, 0.12%, 0.11% and 0.14%, respectively. Once again, the chlorobenzene and chlorophenol (2,4,6-trichlorophenol, 1,2,4-trichlorobenzene, 2-chlorohydroquinone and chloroacetophenone) contents remained unchanged. These results confirmed the bio-accumulative nature of AOX.

### 3.3. Effect of the activated carbon particle size on the adsorption capacity

In the natural environment, pollutants in water are fixed to the sediment. Activated carbon displays the strongest adsorption capacity in sediments [31]. Thus, the adsorption of the total amount of AOXs by sediments was studied using activated carbon as the adsorbent material. The particle size and dosage of the activated carbon, time, pH and temperature are generally investigated in adsorption experiments with activated carbon [32,33]. In this work, activated carbon particles in the range 48–550 µm were employed to investigate the effect of activated carbon particle size on the adsorption capacity. All other experimental conditions were maintained constant (time = 60 min, pH = 3, temperature = 35°C and activated carbon dosage = 1.5 g l^−1^).

**Figure 3.**
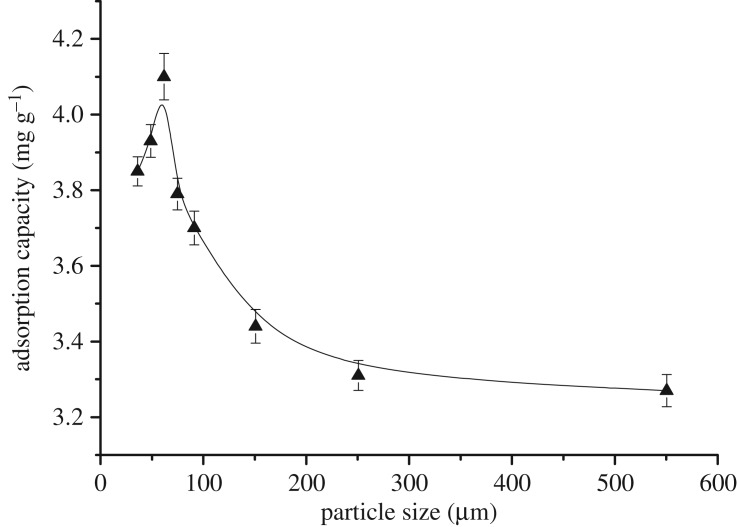
Effect of the activated carbon particle size on the adsorption capacity.

Figure 3 reveals that the AOX content increased from 3.85 mg g^−1^ to 4.10 mg g^−1^ (6.49% increase) as the particle size was increased to less than 62 µm. Subsequently, as the particle size was increased further (greater than 62 µm), the AOX content decreased with an increase in particle size until it became stable at sizes greater than 500 µm. This was attributed to more of the microporous structure being exposed when the particle size was small [34], thereby causing the AOX to desorb into the solution during centrifugation. This trend suggests that the maximum amount of activated carbon adsorption (4.10 mg g^−1^) was achieved at a particle size of 62 µm. In addition, the smaller activated carbon particles were not easily recyclable and exhibited large losses during regeneration. Thus, we concluded that the optimal particle size to achieve maximum AOX removal efficiency was 62 µm.

### 3.4. Effect of time on the adsorption capacity

The study of adsorption time mainly comprises the determination of the equilibrium adsorption time. To investigate the effect of adsorption time on the total amount of AOXs removal, adsorption experiments were carried out in the time range 30–150 min. All other experimental conditions were maintained constant (particle size = 62 µm, pH = 3, temperature = 35°C and activated carbon dosage = 1.5 g l^−1^).

**Figure 4.**
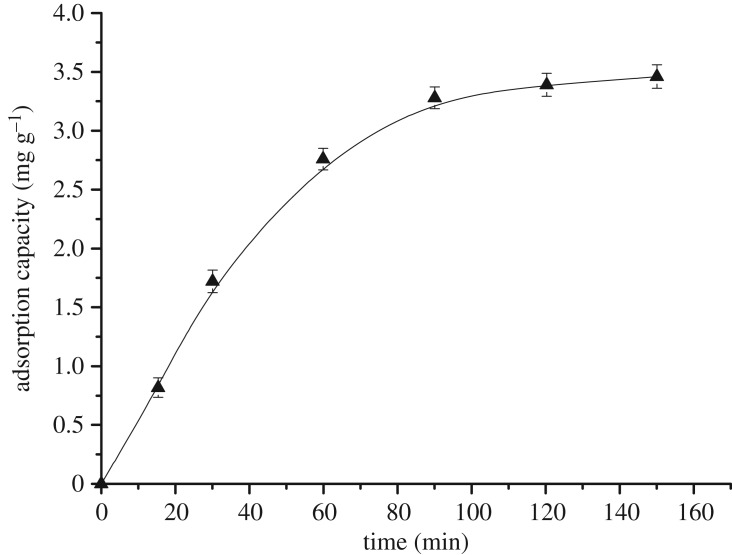
Effect of the time on the adsorption capacity.

Figure 4 illustrates that the adsorption process can be divided into three stages, a fast adsorption phase, a slow adsorption phase and an adsorptive saturation stage. In the first stage, AOX was rapidly adsorbed on the activated carbon and the adsorption capacity increased from 0 to 2.76 mg g^−1^ within 60 min. This was due to the existence of a large number of active adsorption sites on the surface of the activated carbon. In the second stage, between 60 min and 120 min, the adsorption capacity increased from 2.76 mg g^−1^ to 3.39 mg g^−1^ (22.83% increase). As the activated surface-active site becomes occupied by AOX, the compounds begin to diffuse from the outer surface into the adsorbent, thereby affecting the effect of the adsorbent. This results in a decrease in the adsorption rate. Thus, after 120 min, the surface-active sites became completely occupied by AOX. At this stage, both the surface and internal adsorption were almost saturated and the adsorption reached equilibrium. We therefore concluded that the optimal adsorption time was 120 min.

### 3.5. Effect of the pH on the adsorption capacity

The adsorption of organics by activated carbon involves a complex reaction process. This reaction not only has a simple physical role in the adsorption process but is also generally affected by electrostatic, chemical and complexation effects. The pH affects the groups present on the surface of the activated carbon and changes the charge of the surface groups, thus affecting the adsorption process. Hence, the adsorption capacity of the activated carbon exhibits different efficiencies under different pH values. The adsorption experiments were therefore carried out at different pH values in the range 2.1–7.5. The pH of the solution was adjusted by H_2_SO_4_ and NaOH. All other experimental conditions were maintained constant (particle size = 62 µm, time = 120 min, temperature = 35°C and activated carbon dosage = 1.5 g l^−1^).

**Figure 5.**
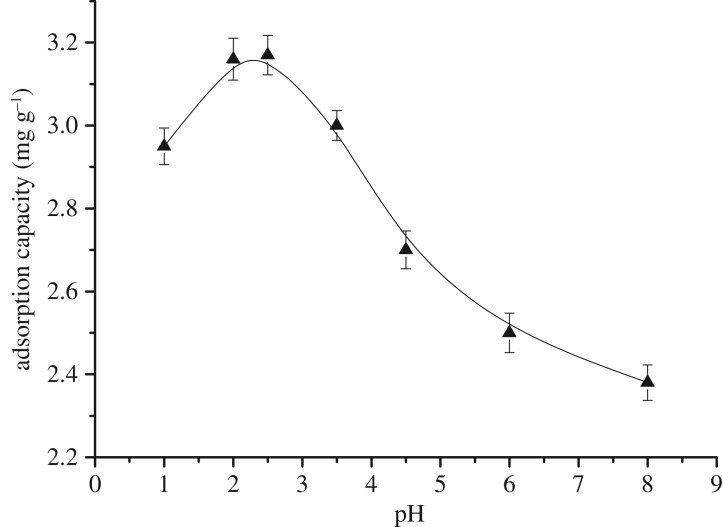
Effect of pH on the adsorption capacity.

Figure 5 reveals that the adsorption capacity increased with a decrease in pH until a maximum adsorption capacity (3.2 mg g^−1^) was observed at pH 2.5. However, this value decreased with a further decrease in pH. At pH 2 and 1, the adsorption capacity was 2.95 mg g^−1^ and 3.16 mg g^−1^, respectively. In fact, the adsorption effect of the activated carbon on an organic pollutant generally decreases with an increase in the pH so that organic pollutants are not easily adsorbed at pH values greater than 8.0 [31]. The ionization of acidic groups, such as carboxyl and phenol hydroxyl groups, on the surface of the activated carbon was inhibited under acidic conditions. Thus, maintaining the state of electrical neutrality is conducive to the adsorption of non-polar organics and phenols. The adsorption effect improved with a decrease in pH. However, the adsorption capacity decreased at pH values less than 2.5. This occurred because the solubility of the adsorbate was affected by the pH of the solution. Thus, the solubility of the organic chloride increased under strong acidic conditions making adsorption by activated carbon difficult. We therefore concluded that the optimal pH to achieve the maximum AOX removal efficiency was 2.5.

### 3.6. Effect of temperature on the adsorption capacity

The reaction temperature is the most basic experimental parameter and affects the efficiency of the activated carbon adsorption process. As an exothermic process, the adsorption process is very sensitive to temperature. Thus, appropriately increasing the temperature facilitates the rapid reaction and adsorption activity. In this study, the adsorption experiments were carried out in the temperature range of 20–50°C. All other experimental conditions were maintained constant (size = 62 µm, time = 120 min, pH = 2.5 and activated carbon dosage = 1.5 g l^−1^).

**Figure 6.**
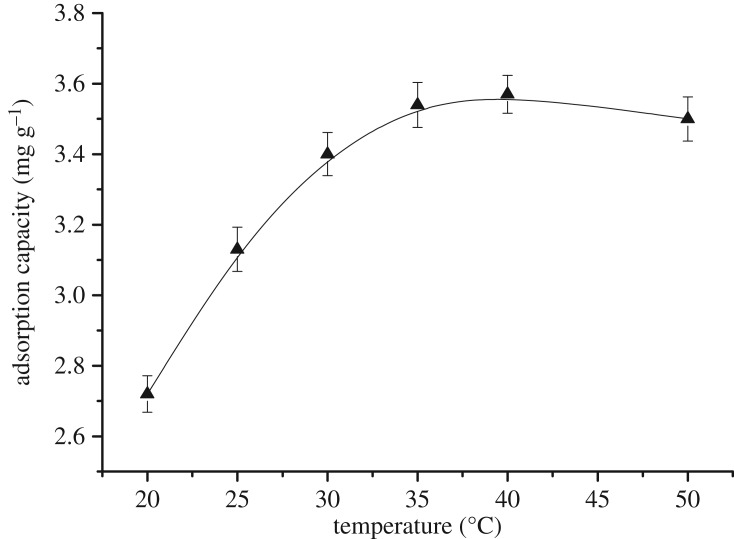
Effect of the temperature on the adsorption capacity.

Figure 6 reveals that a 30% increase in the adsorption capacity occurred as the temperature was increased from 20°C to 35°C (2.72 mg g^−1^ to 3.54 mg g^−1^, respectively). As the temperature was increased further, the adsorption capacity increased slowly until the maximum adsorption capacity (3.6 mg g^−1^) was reached at 40°C. The adsorption capacity was slightly reduced at 50°C (3.50 mg g^−1^), indicating that the dynamic equilibrium of the adsorption process was destroyed and the desorption effect was greater than that of the adsorption [35]. We therefore concluded that the optimal process temperature was 40°C.

### 3.7. Effect of the activated carbon dosage on the adsorption capacity

Adsorption experiments were carried out at different activated carbon dosages in the range 0.25–2.75 g l^−1^. All other experimental conditions were maintained constant (particle size = 62 µm, time = 120 min, pH = 2.5 and temperature = 40°C).

**Figure 7.**
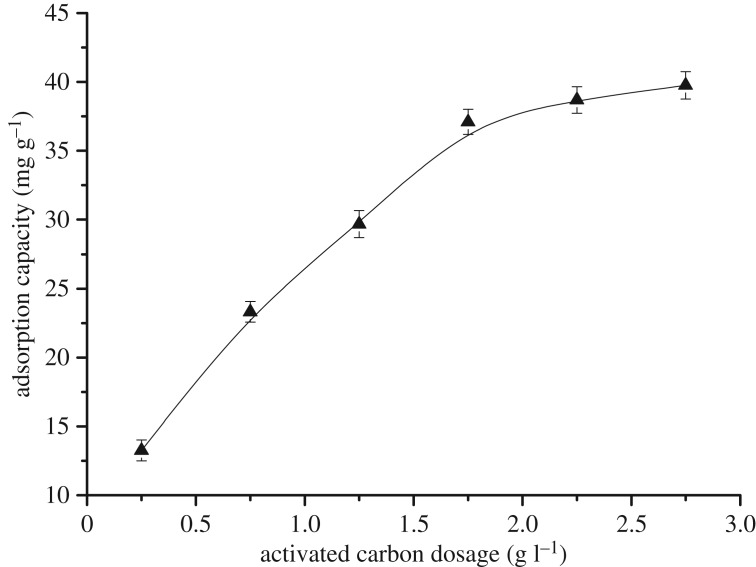
Effect of the activated carbon dosage on the adsorption capacity.

Figure 7 reveals that the activated carbon adsorption process was divided into two stages. A rapid growth stage occurred when the activated carbon dosage was less than 1.75 g l^−1^, whereby the adsorption capacity increased from 13.25 mg g^−1^ to 37.10 mg g^−1^. These results can be attributed to an increase in the unsaturated adsorption sites, surface area and diffusion length. The gradual stage occurred when the dosage was greater than 1.75 l^−1^. In this stage, adsorption only increased from 37.10 mg g^−1^ to 39.75 mg g^−1^, indicating that equilibrium was approached. This occurred because most of the AOX was adsorbed on the surface of the activated carbon. Thus, the active sites on the surface were occupied and further adsorption was inhibited [36]. We therefore concluded that the optimal activated carbon dosage to achieve maximum AOX removal efficiency was 1.75 g l^−1^. At the end of the experiments, the following optimum conditions for the removal of AOX were established: particle size = 62 µm, time = 120 min, pH = 2.5, temperature = 40°C and activated carbon dosage = 1.75 g l^−1^.

### 3.8. Adsorption thermodynamics

The thermodynamics of AOX adsorption by activated carbon was studied under different temperatures. The results are presented in table 2. Δ*G*°, Δ*H*° and Δ*S*° were calculated from equations (3.1)–(3.3).

3.1 $$\Delta G^{\circ }=-RT\ln K_{c},$$

3.2 $$K_{c}=\frac{q_{e}}{C_{e}}$$

3.3 $$\;\mathrm{and}\;\ln K_{c}=\frac{-\Delta H^{\circ }}{RT}+\frac{\Delta S^{\circ }}{R},$$

where *q*_e_ is the adsorption capacity (mg g^−1^) and *C*_e_ is the AOX concentration after adsorption (mg l^−1^).

**Table 2.** Results of adsorption thermodynamic experiment.

	20°C	25°C	30°C	35°C	40°C
*C*_e_ (mg l^−1^)	10.61	8.79	6.81	5.29	4.83
*q*_e_ (mg g^−1^)	26.93	30.12	33.58	36.24	37.04
Δ*G*^0^ (kJ mol^−1^)	−2.27	−3.05	−4.02	−4.93	−5.30
Δ*H*^0^ (kJ mol^−1^)			41.50		
Δ*S*^0^ (J mol^−1^ K^−1^)			149.30		

The adsorption thermodynamics parameters Δ*G*° < 0, Δ*H*° > 0 and Δ*S*° > 0 suggest that the adsorption was a spontaneous entropy increasing process of heat absorption. The results revealed that AOX adsorption by activated carbon was a chemical adsorption process.

### 3.9. GC-MS analysis

The chemical compositions of the bleaching effluent with and without adsorption are presented in table 3. In untreated wastewater, 65% of the chemical composition comprised chlorophenols such as 4,6-dichlororesorcinol, 2,4,6-trichlorophenol and 2-chloro-1,4-benzenediol, while 20% comprised macromolecular chlorides such as 2-chlorocyclohexanone, 4-amino-2-chlorobenzoic acid, 5-chloro-2,1,3-phenoxydiazole, 6-chloropurine and 3,6-dichloropyridazine. Small amounts of micromolecule chlorides, such as methyl dichloroacetate, 1,1,3,3-tetrachloroacetone and 1,1,4-trichlorobutadiene, were also present in the bleaching wastewater. Most chlorophenols were completely eliminated with activated carbon adsorption and only a small amount of 2-chloro-1,4-benzenediol remained. The macromolecular chloride content was also greatly reduced and the 1,1,3-trichloroacetone, 6-chloropurine and 3,6-dichloropyridazine contents decreased by 88.63%, 83.33% and 75.59%, respectively. Moreover, 2-chlorocyclohexanone, 4-amino-2-chlorobenzoic acid and 5-chloro-2,1,3-phenoxydiazole were completely eliminated. Activated carbon also displayed a good removal effect on micromolecule chlorides. Thus, 1,1,3,3-tetrachloroacetone decreased by 92.67%, while 1,1,4-trichlorobutadiene was completely eliminated. These results suggest that activated carbon displays high adsorption efficiency towards AOX.

**Table 3.** Chemical composition analysis of bleaching effluent with or without adsorption.

		content (%)	
retention time (min)	designation	without adsorption	with adsorption
3.206	methyl dichloroacetate	0.03	0.02
6.5	1,1,3-trichloroacetone	0.44	0.05
8.114	1,1,3,3-tetrachloroacetone	1.5	0.11
8.334	2-chlorocyclohexanone	0.28	/
13.118	4,6-dichlororesorcinol	0.5	/
14.922	4-amino-2-chlorobenzoic acid	0.2	/
16.673	2,4,6-trichlorophenol	0.15	/
20.175	2-chloro-1,4-benzenediol	2.57	0.13
21.498	5-chloro-2,1,3-phenoxydiazole	0.25	/
22.092	6-chloropurine	0.84	0.14
34.918	1,1,4-trichlorobutadiene	0.78	/
35.322	3,6-dichloropyridazine	1.27	0.31

## 4. Conclusion

Activated carbon was used to simulate the adsorption of AOX by sediments. The AOX volatilization and degradation processes were studied to exclude their effect on the adsorption process. Micromolecule chlorides were more easily volatilized and degraded than chlorobenzene and chlorophenol. The adsorption of activated carbon to AOX in bleaching wastewater was also studied and the optimum process conditions were elucidated. The chemical compositions of the bleaching effluent with and without adsorption were analysed by GC-MS. The results revealed that activated carbon exhibits good adsorption capacity towards AOX.

## Supplementary Material

ESM for Fig. 1 and 2

## Supplementary Material

ESM for Fig. 3, 4, 5, 6 and 7

## Data accessibility

The datasets supporting this article have been uploaded as part of the manuscript and electronic supplementary material.
