# Supplementary material for: Adsorptive removal of adsorbable organic halogens by activated carbon
Source: R Soc Open Sci. 2018 Dec 5;5(12):181507. doi: 10.1098/rsos.181507 (PMC6304120; doi:10.1098/rsos.181507)
Supplement: ESM for Fig. 1 and 2 [file rsos181507supp1.docx]

Raw data of AOX volatilization and photodecomposition

ESM for Fig. 1

| Time (min) | AOX formation (mg/L) | Error (mg/L) |
| --- | --- | --- |
| 0 | 25.98 | 1.30 |
| 5 | 24.70 | 1.19 |
| 10 | 24.30 | 1.02 |
| 15 | 25.28 | 1.14 |
| 20 | 23.53 | 1.08 |
| 25 | 22.61 | 1.02 |
| 30 | 22.33 | 0.94 |
| 35 | 22.24 | 1.02 |
| 40 | 22.09 | 0.93 |
| 45 | 21.97 | 1.05 |

ESM for Fig. 2

| Time (min) | AOX formation (mg/L) | Error (mg/L) |
| --- | --- | --- |
| 0 | 25.98 | 1.30 |
| 5 | 25.33 | 1.22 |
| 10 | 24.81 | 1.14 |
| 15 | 24.18 | 1.09 |
| 20 | 23.80 | 1.00 |
| 25 | 23.62 | 1.06 |
| 30 | 23.61 | 1.06 |
| 35 | 23.56 | 1.06 |
| 40 | 23.53 | 1.13 |
| 45 | 23.50 | 1.05 |
