# Supplementary material for: Adsorptive removal of adsorbable organic halogens by activated carbon
Source: R Soc Open Sci. 2018 Dec 5;5(12):181507. doi: 10.1098/rsos.181507 (PMC6304120; doi:10.1098/rsos.181507)
Supplement: ESM for Fig. 3, 4, 5, 6 and 7 [file rsos181507supp2.docx]

Effect of active carbon particle size, pH, temperature, dosage, and time on the adsorption capacity

ESM for Fig. 3

| Activated carbon particle size (μm) | Adsorption capacity (mg/g) | Error (mg/L) |
| --- | --- | --- |
| 39.06 | 3.85 | 0.19 |
| 48.79 | 3.93 | 0.18 |
| 61.76 | 4.1 | 0.2 |
| 74.87 | 3.79 | 0.17 |
| 91.09 | 3.7 | 0.17 |
| 150.78 | 3.44 | 0.16 |
| 250.69 | 3.31 | 0.16 |
| 550.43 | 3.27 | 0.15 |

ESM for Fig. 4

| Time (min) | Adsorption capacity (mg/g) | Error (mg/L) |
| --- | --- | --- |
| 0 | 0 | 0 |
| 15 | 0.82 | 0.04 |
| 30 | 1.72 | 0.08 |
| 60 | 2.76 | 0.14 |
| 90 | 3.28 | 0.16 |
| 120 | 3.39 | 0.16 |
| 150 | 3.46 | 0.17 |

ESM for Fig. 5

| pH | Adsorption capacity (mg/g) | Error (mg/L) |
| --- | --- | --- |
| 1.0 | 2.95 | 0.15 |
| 2.0 | 3.16 | 0.15 |
| 2.5 | 3.17 | 0.15 |
| 3.5 | 2.98 | 0.14 |
| 4.5 | 2.71 | 0.11 |
| 6.0 | 2.5 | 0.12 |
| 8.0 | 2.38 | 0.11 |

ESM for Fig. 6

| Temperature (℃) | Adsorption capacity (mg/g) | Error (mg/L) |
| --- | --- | --- |
| 20 | 2.72 | 0.13 |
| 25 | 3.13 | 0.12 |
| 30 | 3.40 | 0.14 |
| 35 | 3.54 | 0.17 |
| 40 | 3.57 | 0.16 |
| 50 | 3.50 | 0.17 |

ESM for Fig. 7

| Activated carbon dosage (g/L) | Adsorption capacity (mg/g) | Error (mg/L) |
| --- | --- | --- |
| 0.25 | 13.25 | 0.60 |
| 0.75 | 23.32 | 1.12 |
| 1.25 | 29.68 | 1.39 |
| 1.75 | 37.10 | 1.56 |
| 2.25 | 38.69 | 1.59 |
| 2.75 | 39.75 | 1.63 |
